# Chestnut tannin extract modulates growth performance and fatty acid composition in finishing Tan lambs by regulating blood antioxidant capacity, rumen fermentation, and biohydrogenation

**DOI:** 10.1186/s12917-023-03870-3

**Published:** 2024-01-10

**Authors:** Changpeng Gao, Mingjiang Qi, Yuxiang Zhou

**Affiliations:** College of Animal Science and Technology, Ningxia University, Yinchuan, China

**Keywords:** Chestnut tannin extract, Plasma metabolites, Rumen fermentation, Growth performance, Fatty acids

## Abstract

Tannins as plant extracts have emerged as promising and potential alternatives for antibiotics in modern livestock cultivation systems. This study investigates the effect of dietary chestnut tannin extract (CTE) in finishing Tan lambs. Twenty-seven male Tan lambs were randomly divided into three groups: (1) control group (CON; basal diet); (2) low-dose CTE group (LCTE; basal diet + 2 g/kg CTE, dry matter [DM] basis); (3) high-dose CTE group (HCTE; basal diet + 4 g/kg CTE, DM basis). The HCTE group exhibited markedly higher average daily gain (ADG) and DM intake than CON (*P* < 0.01). The ruminal total volatile fatty acid concentration increased linearly with increasing CTE supplementation (*P* < 0.01), while the opposite trend was observed for butyrate molar proportion (*P* < 0.01). Upon increasing CTE dosage, plasma glucose, high-density lipoprotein cholesterol, glutathione peroxidase, and superoxide dismutase content increased linearly (*P* < 0.05), whereas low-density lipoprotein cholesterol and urea nitrogen decreased linearly or quadratically (*P* < 0.05), respectively. A linear increase was also observed in ruminal t6 C18:1 and t9, c12 C18:2 proportions (*P* < 0.01), and plasma C18:2n-6 and n-6 polyunsaturated fatty acids proportions with increased CTE supplementation (*P* < 0.01). In the longissimus dorsi muscle, the atherogenic index decreased linearly (*P* < 0.05), while c11 C18:1 and C20:5n-3 increased linearly (*P* < 0.05). Moreover, c9, t11 conjugated linoleic acids proportion increased in subcutaneous fat with CTE supplementation (*P* < 0.01). In conclusion, Dietary CTE enhances the ADG of finishing Tan lambs in a dose-dependent manner, modulates plasma metabolites and antioxidant capacity, and improves rumen fermentation and body fatty acid composition. These results provide a reference for the rational application of CTE in ruminant production.

**Supplementary Information:**

The online version contains supplementary material available at 10.1186/s12917-023-03870-3.

## Introduction

Recently, the growing awareness that health is highly impacted by diet has led to a surge of interest in supplementing ruminant nutrition with natural additives. Consequently, tannins as plant extracts have emerged as promising and potential replacements for antibiotics in modern livestock cultivation systems [1]. Tannins are a class of phenolic compounds secreted by plants for self-protection [2]. Based on their molecular structure and size, tannins are categorized as either hydrolyzable tannins (HT) or condensed tannins (CT) [3]. However, the original belief that tannins are anti-nutrients [4], has greatly hindered their utilization as dietary supplements. It is commonly held that excessive consumption of plant tannins by animals can result in adverse effects, including lower dry matter intake (DMI) and nutrient digestibility [5]. Meanwhile, recent research has demonstrated that ingesting the appropriate quantities of tannins benefits ruminant production [6, 7]. Moreover, although multiple available plant extracts offer different types of tannins, chestnut (*Castanea sativa*) extract, which provides an abundant supply of HT, has been progressively selected as an additive for ruminant diets in research. As reported by Aguerre et al. [8], adding a mixture of 0.45% chestnut and quebracho tannins lowers feed efficiency in dairy cows while increasing milk protein content. Moreover, Taha et al. [9] reported that adding chestnut tannin extract (CTE) to ryegrass silage reduces ammonia nitrogen (NH_3_-N) concentration in lamb rumen. In contrast, Mergeduš et al. [10] discovered that supplementing CTE with a low-protein diet elevates growth performance and nutrition efficiency in bulls, without affecting slaughter performance or meat quality. The differences in these findings could result from the tannin dosage, source, type, animal species, health status, and feed composition [11]. Nevertheless, the precise effect of CTE on the fatty acid profile, rumen hydrogenation, and blood metabolites in sheep remains unclear.

Tan sheep is a unique breed of sheep native to China, predominantly found in the semi-arid desert regions of the Ningxia Hui Autonomous Region. The animal is well-known for its tender meat, delicious flavor, and minimal gamey taste [12]. Therefore, the purpose of this study is to explore how growth performance, fatty acid composition, ruminal characteristics, and plasma metabolites are impacted in finishing Tan lambs supplemented with CTE. We hypothesize that supplementing CTE will improve fatty acid composition, antioxidant capacity, and rumen fermentation.

## Materials and methods

### Ethics statement

All experimental procedures in this study were approved by the Ethics Committee of Ningxia University (protocol NO. NXU-2020-028) and conducted in accordance with ARRIVE guidelines and regulations.

### Animals, experimental design, and diets

The resource equation method was utilized for sample size calculation. According to this method, the value of the error degrees of freedom (DF) in the analysis of variance should lie between 10 and 20 [13]. DF can be measured by the following formula:

DF = total number of animals − total number of groups (1).

When DF = 20 and the total number of groups = 3, the total number of animals = 23. Since 23 is not divisible by 3, the sample size for each group was chosen to be 8. Considering the expected 10% attrition of animals, the corrected sample size was calculated using the following equation:

corrected sample size = sample size/[1− (% attrition/100)] (2).

The calculation gave the corrected sample size of approximately 9, so the corrected total number of animals was 9 × 3 = 27.

This study was conducted at the commercial sheep farm of Ningxia Nongken Co., Ltd. (Yinchuan, China). A total of 27 healthy male Tan lambs, aged 3.00 ± 0.40 months, and weighing 18.77 ± 1.70 kg, were randomly assigned to three groups (*n* = 9) via the PLAN procedure of SAS 9.3 (SAS Institute Inc., Cary, USA): (1) control group (CON; basal diet); (2) low-dose CTE group (LCTE; basal diet + 2 g/kg CTE, dry matter [DM] basis); (3) high-dose CTE group (HCTE; basal diet + 4 g/kg CTE, DM basis). The CTE was provided by Silvateam Technology Co., Ltd. (Guangzhou, China) and contained 75% HT. The CTE was incorporated into the total mixed ration (TMR). All lambs were housed in separate fenced pens (1 m × 2 m) with free access to water and fed TMR twice per day, at 07:00 and 17:00. The entire experimental period lasted 70 d, with the first 10 d dedicated to adaption. The ingredients, chemical composition, and fatty acid profile of TMR are listed in Table 1.

**Table 1:** The ingredients, chemical composition, and fatty acid profile of the basal diet

Item	Content
Ingredients, % dry matter (DM)	
Alfalfa hay	29.8
Corn silage	15.2
Corn	29.5
Soybean meal	14.6
Wheat bran	6.13
Premix^1^	4.90
**Nutrient levels, % DM**	
Crude protein	12.4
Phosphorus	0.36
Calcium	0.52
Acid detergent fiber	21.4
Neutral detergent fiber	32.7
**Fatty acid profile, g/100 g fatty acid**	
C16:0	14.8
C18:0	3.48
C18:1	25.0
C18:2n-6	47.2
C18:3n-6	3.42
C18:3n-3	2.15
C20:0	0.36

^1^ Supplied per kilogram of diet: vit. A 200 kIU, vit. D3 40 kIU, vit. E 1 000 mg, nicotinic acid 1 200 mg, biotin 70 mg, Fe 1 300 mg, Mn 1 000 mg, Co 12 mg, Zn 1 250 mg, Se 8.5 mg

### Sample collection

During the experiment, feed samples were collected weekly for further analysis. Daily feed intake was recorded and the lambs were weighed before receiving their morning meal at the beginning and end of the trial. On d 0, 30, and 60, blood samples were taken via jugular vein and transferred into sodium heparin tubes (Huabo Medical Products Co., Ltd., Heze, China) before morning feeding, then centrifuged (3,000 × g, 15 min, 4 °C) to obtain plasma, which was frozen at -80 °C. Plasma was collected on d 60 for additional FA analysis.

Rumen fluid was collected on d 0, 30, and 60 from each animal through the stomach tube (Anscitech Ltd., Winnipeg, Canada) before morning feeding. Before and after each sample collection, both the inside and outside of the stomach tube were washed with warm water. Salivary contamination rendered the preliminary 25 mL of rumen fluid ineffectual. Rumen fluid pH was determined with a handheld tester (pH828, Kexin Instrument Co., Ltd., Suzhou, China). The rumen fluid sampled on d 0 and 30 was filtered through four layers of sterile gauze before being separated into two equal portions and snap-frozen in liquid nitrogen for NH_3_-N and volatile fatty acid (VFA) analysis. The rumen fluid sampled on d 60 was filtered through four layers of sterile gauze, divided into three equal aliquots, and fast-frozen under liquid nitrogen for subsequent examination of NH_3_-N, VFA, and fatty acids. Following transfer to the lab, all samples were stored in a freezer (Midea Group Co., Ltd., Foshan, China) at -80 °C.

At the end of the feeding trial, all lambs were fasted for 12 h and slaughtered humanely by electrical stunning in the local commercial abattoir (Yinchuan, China). After slaughter, subcutaneous fat and the longissimus dorsi muscle were collected and stored at -80 °C to determine the fatty acid profile.

### Feed chemical analysis

The feed chemical content is presented in Table 1. The analytical approaches and processes have been publicly reported by Liu et al. [14].

### Plasma metabolite and rumen fermentation parameter analysis

Plasma samples’ triglycerides (TG), glucose (GLU), urea nitrogen (BUN), cholesterol (CHOL), total protein (TP), albumin (ALB), low-density lipoprotein cholesterol (LDL-C), and high-density lipoprotein cholesterol (HDL-C) were determined by the BS-420 Automatic Biochemistry Analyzer (Mindray Biomedical Electronics Co., Ltd., Shenzhen, China) adopting standard kits (Jiancheng Bioengineering Institute Co., Ltd., Nanjing, China). The difference between the TP content minus ALB represented the globulin (GLB) content. The protocols of each reagent kit (Jiancheng Bioengineering Institute Co., Ltd., Nanjing, China) were followed to determine malondialdehyde (MDA), superoxide dismutase (SOD), glutathione peroxidase (GSH-Px), and total antioxidant capacity (T-AOC). Additionally, ELISA kits (Enzyme-linked Biotechnology Co., Ltd., Shanghai, China), were employed to quantify immunoglobulin M (IgM), immunoglobulin A (IgA), and immunoglobulin G (IgG) levels.

Ruminal NH_3_-N was analyzed using colorimetry (Beckman Coulter DU730 Spectrophotometer, California, USA), following the steps outlined by Broderick and Kang [15]. The VFA was measured utilizing the gas chromatograph (Shimadzu GC-2030, Kyoto, Japan) and a capillary column (AT-FFAP: 0.5 mm × 0.32 mm × 30 m), according to a previously described protocol [16].

### Fatty acid composition

According to the protocol described by Hao et al. [17], fatty acid analysis was conducted on the diets, plasma, rumen fluid, longissimus dorsi muscle, and subcutaneous fat samples. In brief, lipid extraction was carried out using a mixture (1:2) of methanol and chloroform. Transesterification with boron trifluoride etherate yielded fatty acid methyl ester (FAME). A 100% cyanopropyl polysiloxane capillary column (SP-2560, 100 m × 0.25 mm i.d. with 0.20 μm film thickness) was employed to determine FAME utilizing gas chromatography with flame ionization detection (GC-FID) (Shimadzu GC-2030, Kyoto, Japan). Information regarding the chromatographic settings of GC-FID and GC-MS were obtained from Oliveira et al. [18].

### Statistical analysis

All statistical analyses were conducted using SAS 9.3 (SAS Institute Inc., Cary, USA). Data were presented as least-squares mean and standard error of the mean (SEM). Data on ruminal characteristics and plasma metabolites were analyzed via the PROC MIXED procedure. Repetition of measurements was employed in the complete random block design. The model is represented by the following equation:

Yijkl = µ + Gi + Tj + GTij + Bk + eijkl (3).

where Yijkl is the observations of response variables, µ is the overall means value, Gi is the fixed effects of CTE supplementation (i = 3; CON, LCTE, and HCTE), Tj is the time effects (j = 3; 0 d, 30 d, and 60 d), GTij is the interaction between G and T, Bk is the effects of the random block, and eijkl is the residual error. Polynomial contrasts were selected to evaluate the dose-response of CTE (linear or quadratic). To assess the effects of CTE supplementation on growth performance and fatty acid profile, PROC GLM was adopted. The model remained unalterable, except for the removal of block, time, and diet × time. Tukey’s multiple comparison tests were applied to detect differences among groups. Polynomial contrasts were employed to investigate the effect (linear or quadratic) of the CTE dose. GraphPad Prism 9.0 (GraphPad Software Inc., San Diego, USA) was implemented to construct graphics. For all tests, a significance threshold of *P* < 0.05 was selected, whereas 0.05 ≤ *P* ≤ 0.10 indicated a tendency to differ.

## Results

### Growth performance

**Fig. 1:**
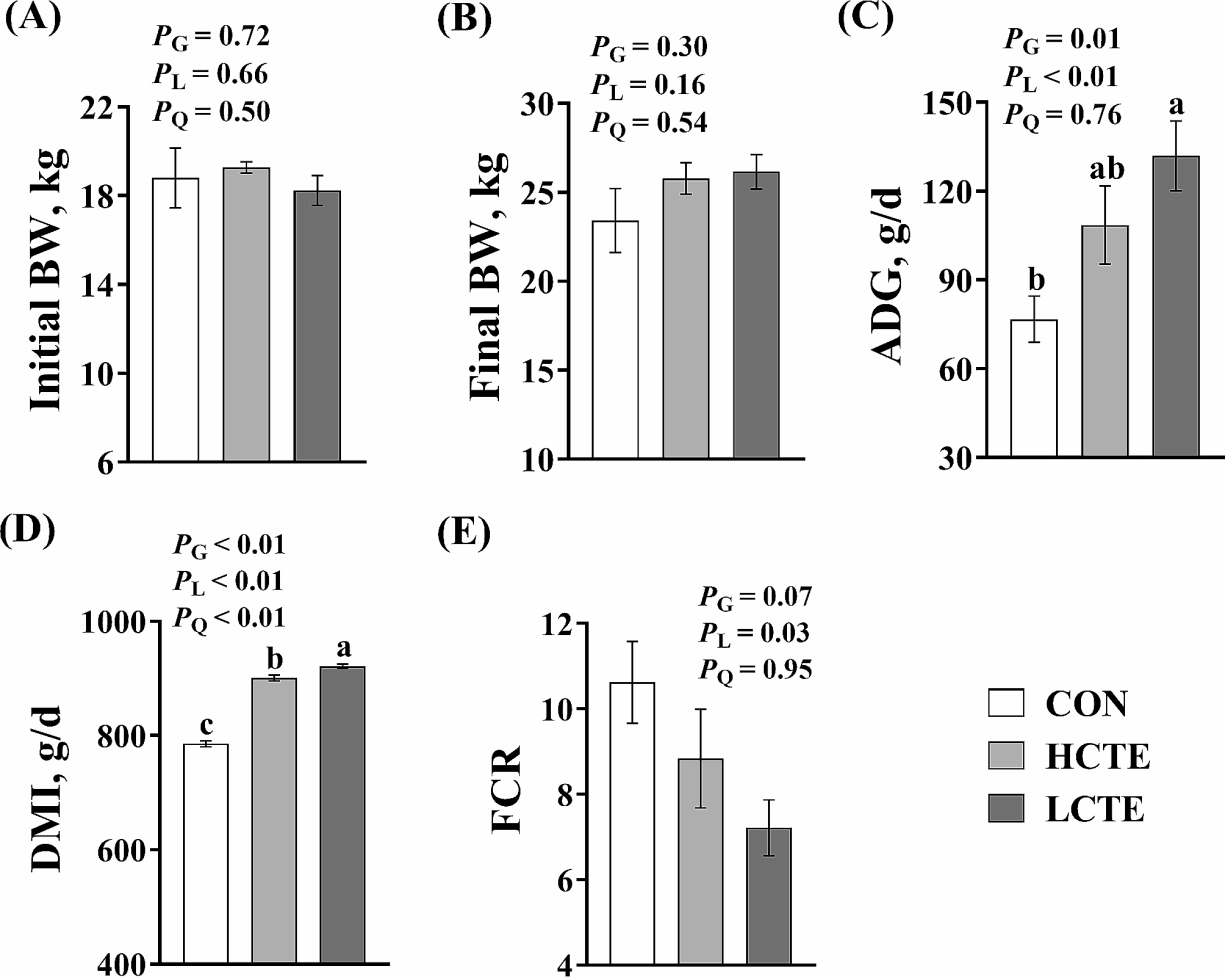
Effect of different chestnut tannin extract doses on growth performance of finishing Tan lambs. **(A)** initial BW = initial body weight; **(B)** final BW = final body weight; **(C)** ADG = average daily gain; **(D)** DMI = dry matter intake; **(E)** FCR = feed conversion ratio. CON = control; LCTE = 2 g/kg chestnut tannin extract; HCTE = 4 g/kg chestnut tannin extract. The effects included group (G) effects, linear (L) effects, and quadratic (Q) effects. ^a, b, c^ Values with distinct letters differ significantly (*P* < 0.05). The error bar represents the standard error of the mean

There were no significant differences in the initial and final body weight (BW) among the three groups (*P* > 0.05; Fig. 1A, B). With increasing supplemental doses of CTE, average daily gain (ADG) and DMI increased linearly (*P* < 0.01; Fig. 1C, D). DMI was higher with CTE supplementation than in the CON group (*P* < 0.01; Fig. 1D), and ADG was greater for the HCTE group than the CON group (*P* ≤ 0.01; Fig. 1C). Furthermore, there was a tendency (*P* = 0.07; Fig. 1E) for feed conversion rate (FCR) to decrease with increasing levels of CTE supplementation.

### Rumen fermentation parameters

**Fig. 2:**
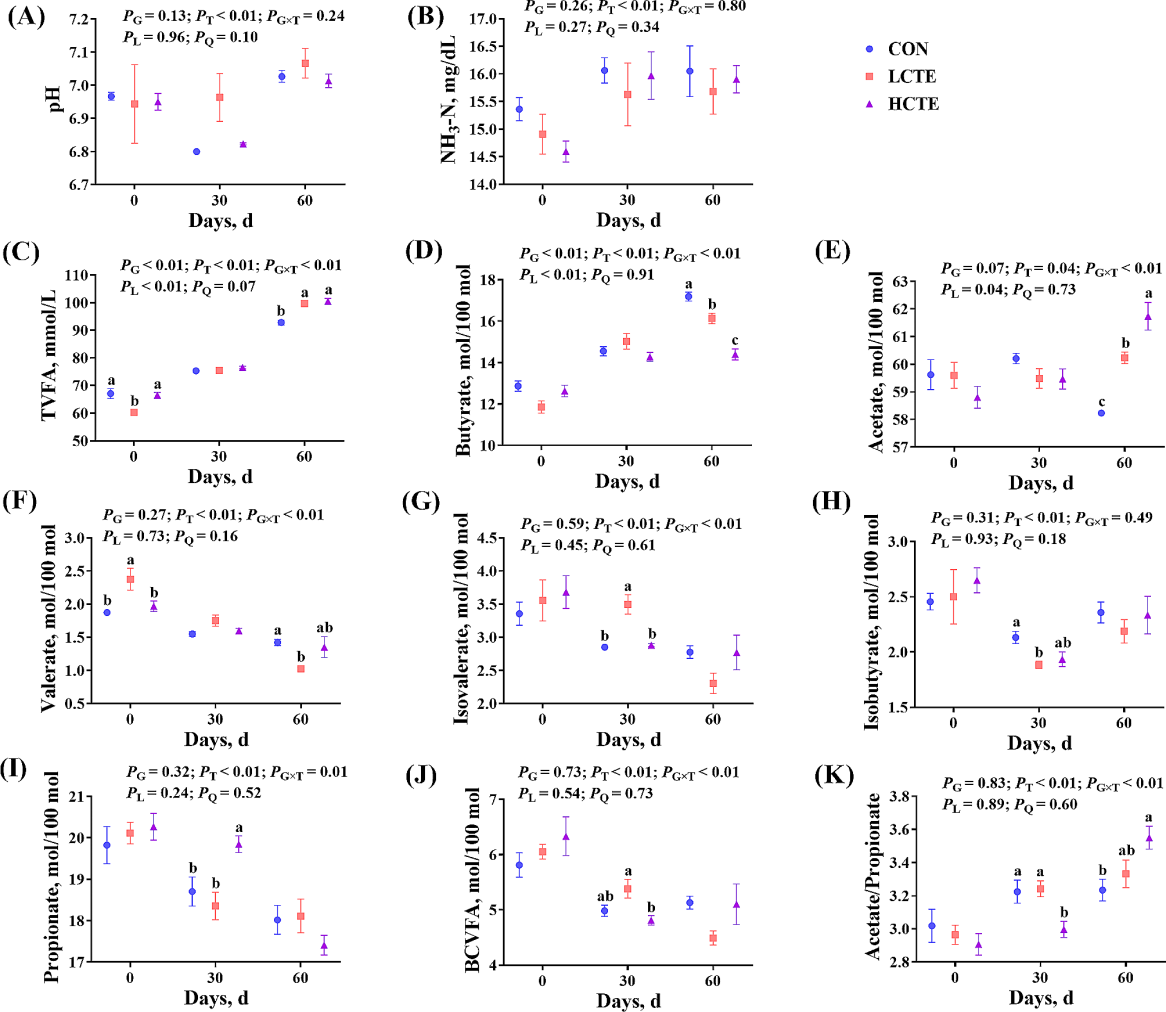
Effect of chestnut tannin extract supplementation after 0, 30, or 60 d on the ruminal fermentation characteristics of finishing Tan lambs. **(A)** pH; **(B)** NH_3_-N; **(C)** TVFA = total volatile fatty acids; **(D)** butyrate; **(E)** acetate; **(F)** valerate; **(G)** isovalerate; **(H)** isobutyrate; **(I)** propionate; **(J)** BCVFA = branched-chain volatile fatty acids; **(K)** acetate/propionate. CON = control; LCTE = 2 g/kg chestnut tannin extract; HCTE = 4 g/kg chestnut tannin extract. The effects included group (G) effects, time (T) effects, and interaction effects between group and time (G × T), as well as linear (L) and quadratic (Q) effects. The mean and standard error of the mean are plotted. Bars with distinct superscripts (a–c) differ significantly (*P* < 0.05)

Rumen pH and NH_3_-N were not affected by CTE (*P* > 0.05; Fig. 2A, B). Meanwhile, the total VFA (TVFA) content increased linearly with an increasing CTE dose (*P* < 0.01; Fig. 2C), and the butyrate molar proportion exhibited the opposite trend (*P* < 0.01; Fig. 2D), but both had interaction effects with group and time (G × T; *P* < 0.01). Acetate exhibited an upward trend (*P* = 0.07; Fig. 2E). Although, on d 0, LCTE had a lower TVFA concentration than CON or HCTE (*P* < 0.05; Fig. 2C), a higher molar proportion of valerate (*P* < 0.05; Fig. 2F) was observed. On d 30, compared to the LCTE, the molar proportion of isovalerate was observably higher than those in other groups (*P* < 0.05; Fig. 2G), whereas that of isobutyrate was observably lower than in CON (*P* < 0.05; Fig. 2H). On d 30, the molar proportion of propionate was higher in HCTE than in CON and LCTE (*P* < 0.05; Fig. 2I), while that of branched-chain volatile fatty acids (BCVFA) was higher than in LCTE (*P* < 0.05; Fig. 2J). On d 60, the TVFA concentration and molar proportion of acetate increased for CTE (*P* < 0.05; Fig. 2C, E), whereas acetate/propionate was higher in HCTE than CON (*P* < 0.05; Fig. 2K).

### Plasma biochemical indices, antioxidant capacities, and immune functions

**Fig. 3:**
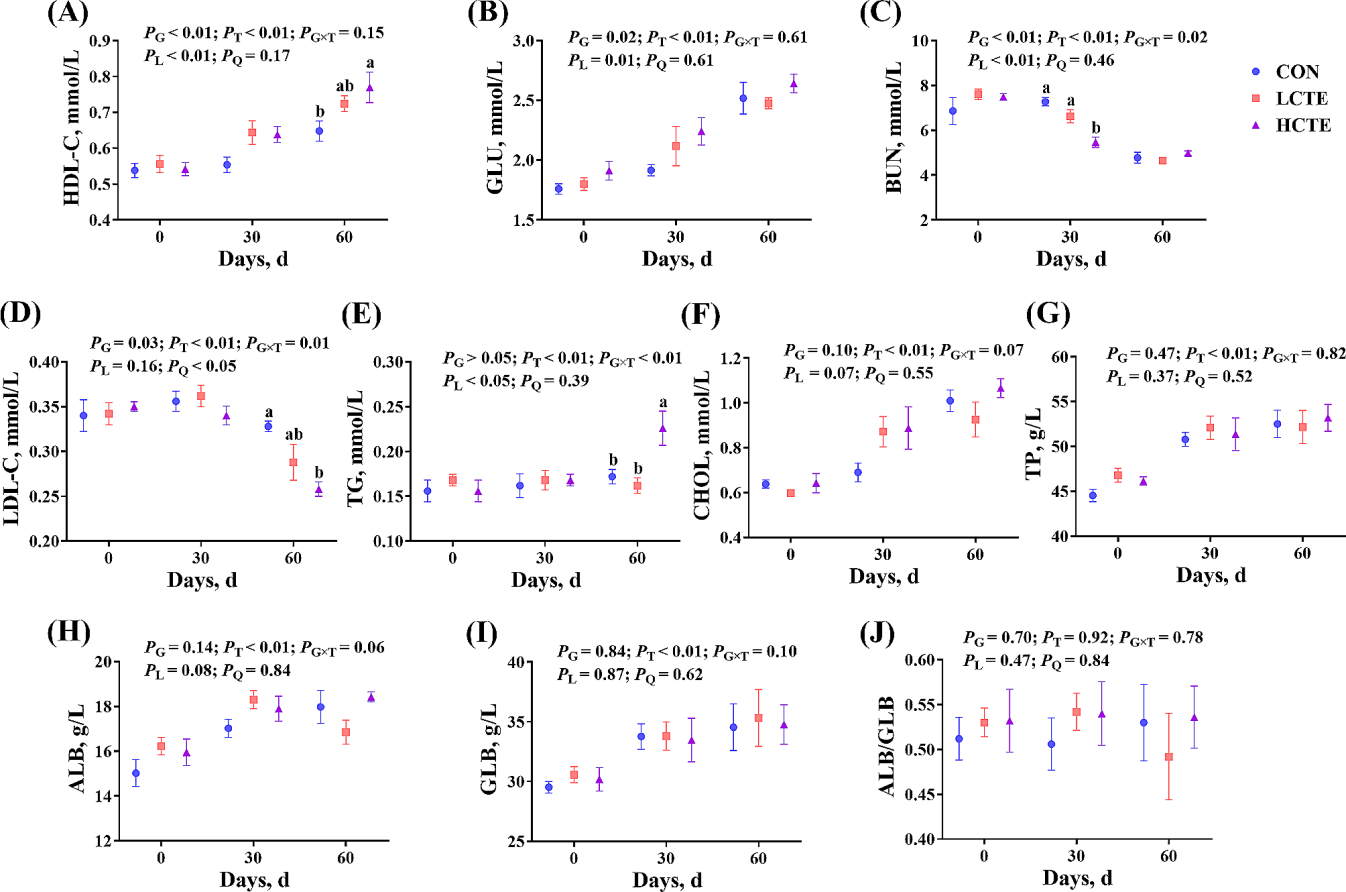
Plasma biochemical indices in finishing Tan lambs fed chestnut tannin extract within three sampling days. **(A)** HDL-C = high-density lipoprotein cholesterol; **(B)** GLU = glucose; **(C)** BUN = urea nitrogen; **(D)** LDL-C = low-density lipoprotein cholesterol; **(E)** TG = triglycerides; **(F)** CHOL = cholesterol; **(G)** TP = total protein; **(H)** ALB = albumin; **(I)** GLB = globulin; **(J)** ALB/GLB = albumin/globulin. CON = control; LCTE = 2 g/kg chestnut tannin extract; HCTE = 4 g/kg chestnut tannin extract. The effects included group (G) effects, time (T) effects, and the interaction effects between group and time (G × T), as well as linear (L) and quadratic (Q) effects. Values are mean ± standard error of the mean. Bars with distinct superscripts (a, b) differ significantly (*P* < 0.05)

Regarding plasma biochemical indices, the contents of HDL-C and GLU increased linearly (*P* ≤ 0.01; Fig. 3A, B), while those of BUN and LDL-C decreased in a linear or quadratic manner (*P* < 0.05; Fig. 3C, D), respectively, with increasing CTE supplementation and study duration. Furthermore, the G × T interaction was identified for BUN and LDL-C (*P* < 0.05). That is a lower BUN content was detected in HCTE compared with CON or LCTE on d 30 (*P* < 0.05; Fig. 3C). Meanwhile, on d 60, the HDL-C and TG contents were higher in HCTE (*P* < 0.05; Fig. 3A, E), whereas LDL-C was lower in HCTE compared with CON (*P* < 0.05; Fig. 3D). Moreover, the contents of CHOL, TP, ALB, GLB, and ALB/GLB were not significantly affected by the CTE (*P* > 0.05; Fig. 3F-J).

**Fig. 4:**
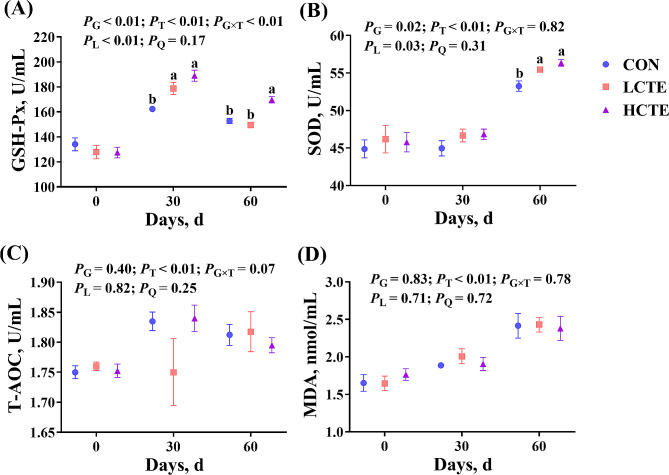
Plasma antioxidant capacity of finishing Tan lambs fed chestnut tannin extract within the three sampling days. **(A)** GSH-Px = glutathione peroxidase; **(B)** SOD = superoxide dismutase; **(C)** T-AOC = total antioxidant capacity; **(D)** MDA = malonaldehyde. CON = control; LCTE = 2 g/kg chestnut tannin extract; HCTE = 4 g/kg chestnut tannin extract. The effects included group (G) effects, time (T) effects, and the interaction effects between group and time (G × T), as well as linear (L) and quadratic (Q) effects. Values are mean ± standard error of the mean. Bars with distinct superscripts (a, b) differ significantly (*P* < 0.05)

**Fig. 5:**
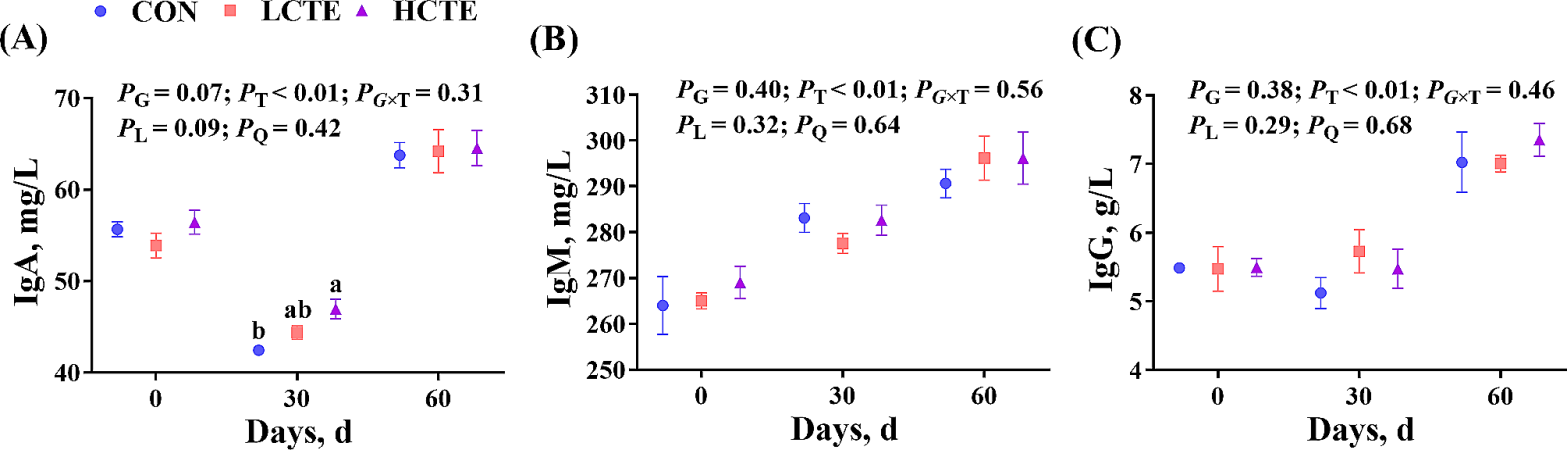
Plasma immune function in finishing Tan lambs fed chestnut tannin extract within the three sampling days. **(A)** IgA = immunoglobulin A; **(B)** IgM = immunoglobulin M; **(C)** IgG = immunoglobulin G. CON = control; LCTE = 2 g/kg chestnut tannin extract; HCTE = 4 g/kg chestnut tannin extract. The effects included group (G) effects, time (T) effects, and the interaction effects between group and time (G × T), as well as linear (L) and quadratic (Q) effects. Values are the mean ± standard error of the mean. Bars with distinct superscripts (a, b) differ significantly (*P* < 0.05)

Regarding plasma antioxidant activity, GSH-Px and SOD contents increased linearly (*P* < 0.05; Fig. 4A, B), while no statistical significances were found in T-AOC and MDA contents (*P* > 0.05; Fig. 4C, D). On d 30, the GSH-Px content in CTE increased than in CON (*P* < 0.05; Fig. 4A), while on d 60, the GSH-Px content was increased in the HCTE group compared with the CON and LCTE groups (*P* < 0.05; Fig. 4A). Moreover, on d 60, the SOD content was greater in the two CTE groups compared with CON (*P* < 0.05; Fig. 4B). Additionally, plasma immune function analysis revealed that IgA content tended to increase (*P* = 0.07; Fig. 5A), but IgM and IgG were not affected by supplementation (*P* > 0.05; Fig. 5B, C).

### Fatty acid profiles in the ruminal, plasma, longissimus dorsi muscle, and subcutaneous fat

The ruminal fatty acid composition is presented in Supplementary file 1. The proportions of t9, c12 C18:2, and t6 C18:1 were higher in HCTE than in the other groups (*P* < 0.01). Simultaneously, t9 C18:1 and t11 C18:1 exhibited increasing tendencies (*P* = 0.08, 0.10, respectively).

In response to CTE supplementation, plasma C18:1, and t11 C18:1 decreased in a linear manner (*P* ≤ 0.01; Supplementary file 2). In contrast, plasma n-6 polyunsaturated fatty acids (PUFA) and C18:2n-6 increased linearly (*P* ≤ 0.01), with both peaking in the HCTE group. Additionally, t11 C18:1 was lower in HCTE than in CON (*P* < 0.05). Following CTE supplementation, c9 C18:1, PUFA, and PUFA/saturated fatty acids (SFA) had a tendency to be higher (*P* = 0.09, 0.08, 0.10, respectively), whereas C15:0 and monounsaturated fatty acids (MUFA)/PUFA exhibited a tendency to be lower (*P* = 0.10, 0.05, respectively).

Table 2 summarizes the fatty acid profile within the longissimus dorsi muscle. With increasing CTE supplementation, C16:1, the atherogenic index (AI), and C22:0 decreased in a linear or quadratic manner (*P* < 0.05), while SFA, the thrombogenic index (TI), and C16:0 tended to decrease (*P* = 0.06, 0.05, 0.08, respectively). However, c11 C18:1 and C20:5n-3 increased linearly (*P* < 0.05), and PUFA/SFA, unsaturated fatty acids (UFA), and UFA/SFA trended upward (*P* = 0.06 for all three). A significant increase in C20:5n-3 and C16:1 content was observed in HCTE compared with CON (*P* < 0.05). Moreover, compared to CON, the CTE group exhibited increased c11 C18:1 (*P* < 0.05) and reduced AI (*P* < 0.05).

**Table 2:** Effect of chestnut tannin extract on the fatty acid profile of longissimus dorsi muscle in finishing Tan lambs (g/100 g of total fatty acids)

Item	CTE Addition			SEM	P-Value		
	CON	LCTE	HCTE		G	L	Q
C14:0	2.29	2.23	2.19	0.044	0.28	0.12	0.80
C14:1	0.08	0.08	0.08	0.002	0.34	0.27	0.34
C15:0	0.59	0.60	0.59	0.012	0.95	0.86	0.79
C15:1	1.66	1.67	1.66	0.020	0.88	0.94	0.63
C16:0	18.28	17.43	17.24	0.313	0.08	0.04	0.40
C16:1	1.91^a^	1.85^ab^	1.77^b^	0.033	0.03	0.01	0.67
C17:0	2.33	2.38	2.34	0.024	0.37	0.77	0.18
C17:1	0.64	0.64	0.62	0.008	0.11	0.06	0.31
C18:0	14.17	14.41	14.47	0.197	0.53	0.30	0.72
C18:1n-9	46.10	46.47	46.63	0.304	0.47	0.24	0.78
t11 C18:1	0.63	0.64	0.65	0.010	0.47	0.25	0.75
t9 C18:1	0.03	0.03	0.03	0.001	0.19	0.08	0.57
c11 C18:1	0.02^b^	0.03^a^	0.03^a^	0.001	0.03	0.03	0.09
C18:2n-3	0.18	0.17	0.18	0.004	0.26	0.20	0.30
C18:2n-6	8.39	8.64	8.79	0.147	0.20	0.08	0.76
c9, t11 CLA	0.15	0.15	0.15	0.001	0.44	0.21	0.97
c9, c12 C18:2	0.08	0.08	0.08	0.002	0.60	0.45	0.50
c10, t12 C18:2	0.02	0.02	0.02	0.001	0.78	0.50	0.88
C18:3n-6	0.15	0.15	0.16	0.003	0.34	0.15	0.92
C18:3n-3	0.22	0.23	0.22	0.004	0.93	0.96	0.72
C20:0	0.08	0.08	0.08	0.001	0.44	0.31	0.45
C21:0	0.23	0.22	0.23	0.004	0.29	0.64	0.14
C20:1	0.11	0.11	0.10	0.001	0.40	0.85	0.19
C20:3n-6	0.02	0.02	0.02	0.000	0.30	0.68	0.14
C20:4n-6	0.80	0.81	0.83	0.014	0.49	0.25	0.92
C20:5n-3	0.27^b^	0.28^ab^	0.29^a^	0.005	0.02	< 0.01	0.59
C22:0	0.101^ab^	0.103^a^	0.098^b^	0.001	0.04	0.10	0.04
C22:6n-3	0.05	0.05	0.05	0.001	0.99	0.94	0.94
C24:0	0.15	0.15	0.16	0.004	0.90	0.69	0.84
C24:1	0.25	0.27	0.25	0.008	0.44	0.92	0.21
SFA	38.23	37.60	37.40	0.231	0.06	0.03	0.46
MUFA	51.43	51.79	51.82	0.276	0.55	0.34	0.63
PUFA	10.34	10.61	10.78	0.164	0.20	0.08	0.80
UFA	61.77	62.40	62.60	0.231	0.06	0.03	0.46
n-6 PUFA	9.37	9.63	9.79	0.161	0.21	0.09	0.79
n-3 PUFA	0.72	0.73	0.74	0.008	0.53	0.27	0.95
MUFA/SFA	1.35	1.38	1.39	0.015	0.17	0.08	0.50
PUFA/SFA	0.27	0.28	0.29	0.005	0.06	0.02	0.62
UFA/SFA	1.62	1.66	1.67	0.016	0.06	0.03	0.45
MUFA/PUFA	4.98	4.89	4.82	0.092	0.49	0.24	0.92
n-6/n-3	12.93	13.18	13.25	0.240	0.62	0.36	0.76
AI	0.45^a^	0.42^b^	0.42^b^	0.007	0.03	0.01	0.37
TI	1.06	1.03	1.02	0.011	0.05	0.02	0.41

CTE = chestnut tannin extract; CON = control; LCTE = 2 g/kg chestnut tannin extract; HCTE = 4 g/kg chestnut tannin extract; CLA = conjugated linoleic acids; SFA = saturated fatty acids; MUFA = monounsaturated fatty acids; PUFA = polyunsaturated fatty acids; UFA = unsaturated fatty acids; n-6/n-3 = n-6 PUFA/n-3 PUFA; AI = atherogenic index; TI = thrombogenic index. The effects included group (G) effect, linear (L) effect, and quadratic (Q) effect. Values are means ± standard error of the mean (SEM). On the same line, data with different superscripts (a, b) represent significant differences (*P* < 0.05)

The fatty acid profile of subcutaneous fat is presented in Supplementary file 3. There was no significant difference in SFA was observed among the three groups (*P* > 0.05), and in PUFA (*P* > 0.05). Although C20:0 and c9, t11 conjugated linoleic acids (CLA) increased linearly (*P* < 0.01), there was also a tendency for a quadratic increase in c9, t11 CLA (*P* = 0.05). In particular, increased C20:0 and c9, t11 CLA content was observed in LCTE and HCTE compared with CON (*P* < 0.01).

## Discussion

In the current study, the ADG of finishing Tan lambs increased with increasing dietary supplementation of CTE, while the FCR showed a decreasing trend, indicating improved growth performance, which was previously reported in ruminant studies [6, 7]. This might be related to the nutritional metabolism of ruminants and the specific effect of tannin. Ruminants can take advantage of their symbiotic association with rumen microbes in terms of digestion, which can generate nutrients from decomposition products [10]. We hypothesized that dietary CTE affected microbial diversity and by effect, nutrient synthesis in the rumen. Because tannin can increase rumen escape protein, it induces an increase in the supply of digestible protein to the small intestine and the absorption of amino acids into the blood, thus improving growth performance [19]. These results are inconsistent with those of Taha et al. [9], who reported that CTE (30 g/kg DM) supplementation negligibly impacted lamb growth performance. The reason for this discrepancy could be associated with the dosage of tannin. Meanwhile, Fiorentini et al. [20] reported that 60–90% of animal performance can be attributed to DMI. Therefore, the nutrition requirements of Tan lambs appear to have greater effects on DMI than tannin. The HCTE group exhibited the best growth performance, which might correspond with its exhibition of the highest DMI. Indeed, DMI increased after CTE supplementation; the reason for this observation cannot be explained by the current study, possibly because dietary palatability was not significantly affected within the range of CTE supplementation, and the threshold of altering lamb feed intake was not achieved. However, tannin-containing feeds might be less palatable due to the binding of tannin to salivary proteins, resulting in an astringent or bitter taste in the mouth of the animal [4], which can negatively affect DMI. Although Serri et al. [21] reported no reduction in DMI in animals fed CTE, Dschaak et al. [22] stated that dairy cows supplemented with quebracho tannin extract exhibited lower DMI. Hence, different tannin concentrations, types, structures, and sources, as well as animal species and basal diets, likely elicit different effects on DMI. We thus recommend further studies on the effect of the range of CTE addition on DMI in the future.

Ruminal pH reflects the internal homeostasis of the rumen ecology. In this study, the pH values for each group differed from d 0 to 60, falling within the typical range for optimal rumen function in ruminants (6.2–7.2) [23]. Wang et al. [24] and Ban et al. [25] obtained similar results in *in-vitro* fermentation and in goats, respectively. Moreover, the rumen NH_3_-N range in this study (14.59–16.06 mg/dL) was within the suitable range for rumen microbial development (3.5–25 mg/dL) [26]. Although CTE supplementation had no apparent influence on ruminal NH_3_-N, the NH_3_-N concentrations of the LCTE and HCTE groups were numerically lower than that of the CON group at d 30 and 60. This was comparable to the results of Dschaak et al. [22], who found that adding quebracho tannin extract (3% DM) slightly reduced ruminal NH_3_-N concentration in dairy cows. The decline in NH_3_-N may have been caused by tannin, which can combine with feed proteins to generate a protein-tannin complex that resists rumen microbial degradation [27], thus reducing NH_3_-N production during rumen fermentation. It is well known that ruminants meet their metabolic protein requirements using two sources (microbial protein and bypass protein). The reduction in NH_3_-N concentration represents not only decreased protein degradation during rumen fermentation, but also improved nitrogen utilization efficiency of rumen microbes [28].

VFA provides more than 70% of the energy requirement for ruminant activity and maintains normal rumen function. The recommended TVFA concentration range for ruminal is 60–150 mmol/L [29]; that within the current study was 60.30–100.06 mmol/L. The addition of CTE increases ruminal TVFA [30]. Ban et al. [25] reported an increase in ruminal TVFA and acetate after giving mangosteen (*Garcinia mangostana L.*) peel with CT-enriched feed to sheep. Moreover, in vivo results indicate that supplementation of HT improves acetate synthesis [31]. These results supported our findings, where an increase in TVFA and acetate indicated enhanced digestion and rumen fermentation of structural carbohydrates, which corresponded to an increase in growth performance. Two factors were responsible for the results. First, HT is further converted into several intermediates in the step-by-step enzymatic reactions of biodegradation, to generate acetate [32]. Second, tannins exhibit a high capacity to inhibit methanogens. When methanogens are suppressed, acetogenesis predominates over methanogenesis, which is more advantageous for acetogens to compete with methanogens for the utilization of H_2_ and CO_2_, ultimately generating acetate in the rumen [33]. Such an increase in acetate production might be the mechanism underlying the reduction in methanogenesis caused by CTE addition. However, given that we did not evaluate methane emission, the precise cause requires further investigation.

The molar proportion of butyrate decreased linearly with CTE supplementation, which agreed with previous results [34]. This change might be associated with tannins’ capacity to alter certain protozoan species, which are the primary butyrate producers, resulting in a drop in ruminal butyrate [31]. CT supplementation reportedly reduced the proportion of isobutyrate and isovalerate in lamb rumen [35], while acacia tannin extract supplementation decreased the proportion of BCVFA during i*n vitro* fermentation [36]. Generally, BCVFA is derived primarily from the catabolism of ruminal branched-chain amino acids; its concentration increases with dietary energy levels [37]. Moreover, CT has a stronger affinity for feed protein than HT, which hinders the deamination of soluble protein in the rumen, as reflected by the decrease in BCVFA [38]. This may account for the lack of significant changes in isobutyrate, isovalerate, or BCVFA in the current study. Teobaldo et al. [39] demonstrated that dietary HT induces more propionate and less valerate production in the rumen of beef cattle. Similarly, HT supplementation decreases acetate/propionate in fermentation [24]. Hence, the absence of changes in propionate, valerate, or acetate/propionate might be due to the CTE level being insufficient to cause these effects. Notably, the variation of each molar proportion of ruminal VFA in small ruminants depends on thermal environmental issues, which may be related to higher temperatures or climactic conditions [40].

Herein, we adopted the CTE supplementation model to evaluate the plasma biochemical indexes of finishing Tan lambs. BUN is regulated by the body’s metabolism of dietary protein and reflects how efficiently animals utilize nitrogen [19]. Therefore, the observed linear decrease in BUN content following CTE supplementation indicates improved nitrogen use. This might be because tannins slow down the liver’s ability to absorb NH_3_-N by inhibiting protein hydrolysis in the rumen. The liver then converts nitrogen into urea, which enters the bloodstream [41]. A previous study found that BUN concentration noticeably decreased with an increase in tannin dosage [42], which is consistent with our results. Moreover, plasma GLU content increased in lambs supplemented with CTE, which agrees with the results of a previous study [43]. The blood GLU level reflects the dynamic energy metabolism balance. Tannins can interfere with the gram-negative microbiome in the rumen, and these bacteria have a lower energy requirement, thus increasing the liver’s ability to synthesize glucose precursors [44], which could explain the higher level of plasma GLU detected in this study. Meanwhile, promoting gluconeogenesis and intestinal glucose absorption, or both, may lead to higher plasma GLU levels [45].

Plasma TG, CHOL, HDL-C, and LDL-C contents are considered indicators of lipid metabolism in animals [46]. We found that CTE had no discernible impact on serum CHOL or TG concentrations, which agreed with the findings of Moheghi et al. [47]. The transport of lipids from secretory organs, such as the liver and intestine, to peripheral tissues depends on plasma lipoproteins. Among these, HDL-C can transport excess CHOL from the blood to the liver for catabolism, while LDL-C performs the opposite function [48]. Indeed, tannins can considerably alter blood lipoprotein levels [49]. Similarly, the current research confirmed the beneficial implications of CTE addition for lipid metabolism in Tan lambs. Given that HDL-C increased linearly, while LDL-C decreased quadratically following the addition of CTE over time, we speculate that 3-hydroxy-3-methyl glutaryl coenzyme A reductase—a crucial regulator of CHOL synthesis—may have a critical role in the CTE regulatory effect. Thus, the sn-2 acyl chains of HDL-C phosphatidylcholine are catalytically transferred to the 3-hydroxyl group of unesterified CHOL [50]. Moreover, LDL-C content is associated with endothelial dysfunction and abnormal inflammation. This experiment was carried out under normal conditions; hence, the low plasma LDL-C content was in line with expectations. TP, ALB, and GLB are primarily synthesized in the liver and can reflect animal protein metabolism [51]. In the current study, plasma TP, ALB, GLB, and the ALB: GLB ratio did not significantly differ among groups at d 0 to 60 and were all within normal limits. This indicated that the addition of CTE had no detrimental influence on liver metabolism in Tan lambs.

Numerous physiological processes involved in animal growth result in the generation of free radicals, which, if not scavenged, may initiate oxidative stress [52]. Previous studies have reported the antioxidant capacity of tannins [2, 53]. However, the biological processes underlying how tannins respond to antioxidant enzymes are not fully understood. Nevertheless, the addition of CTE to the diets of transition cows increases their plasma SOD and GSH-Px activities [54]. Correspondingly, in the present study, CTE considerably improved plasma SOD and GSH-Px contents of Tan lambs in a dose-dependent manner. This might be caused by the activation of the nuclear factor erythroid 2-related factor 2 signaling pathway by tannins, resulting in the upregulation of genes encoding antioxidant enzymes [55]. Additionally, the redox characteristics of the chemical structure of tannins might activate antioxidant defenses to eliminate free radicals. Hence, CTE supplementation might be a workable strategy to improve the antioxidant status of lambs during the finishing period.

Immunoglobulins are involved in specific and non-specific immune responses against different inflammatory conditions [56]. In this study, IgM and IgG concentrations were unaffected by CTE; however, IgA concentrations tended to increase. The increase in IgA within the CTE-supplemented group might indicate increased resistance to pathogens, as IgA agglutinates pathogens and enhances adhesion to bacteria. These results differed slightly from those of studies on buffalos [57] and goats [58]. This discrepancy could be due to tannins having diverse effects and mechanisms on the immune function of various ruminants.

Earlier research on ruminal fatty acid suggested that tannins might alter ruminal biohydrogenation (BH) patterns, which could lead to a decrease in C18:0 and an increase in BH intermediates, including trans-C18:1 and c9, t11 CLA [59]. We found that t6 C18:1 increased linearly with increasing CTE addition, while t11 C18:1 or t9 C18:1 tended to increase. As isomers of trans-C18:1, elevated t6 C18:1, t9 C18:1, and t11 C18:1 indicate incomplete hydrogenation of rumen UFA. The results of this study were also indirectly confirmed by the significantly higher ruminal t9, c12 C18:2 in HCTE than in the other groups. In addition, previous research found that c9, t11 CLA and t11 C18:1 detected in the rumen are only produced by incomplete hydrogenation [60]. These results show that the first step of BH appears to be less affected by tannins than the subsequent reactions. The formation of t11 C18:1 is known to be conducted by class I bacteria, while the subsequent pathway of BH is conducted by class II bacteria. Therefore, it can be hypothesized that tannin has a greater influence on class II bacteria than on class I bacteria. Moreover, it was reported that rumen protozoa can convert c9, c12 C18:2 to c9, t11 CLA, but not c9, t11 CLA to t11 C18:1 [61]. Therefore, c9, t11 CLA is synthesized by bacteria and protozoa, while t11 C18:1 is derived from only bacteria. This might explain why the last step of BH is more affected by tannins than the previous step. However, further study is required to provide more information.

The transfer of long-chain fatty acids depends on the allocation of absorbed fatty acids between very low-density lipoprotein and chylomicron, and their binding to TG and CHOL. Although blood fatty acid composition reflects rumen fatty acid composition to a certain degree, the results have been variable. n-6 PUFA is honored as a functional fatty acid due to its anticancer and hypolipidemic properties [62]. In this study, the increase in plasma n-6 PUFA and C18:2n-6 implies that CTE supplementation might inhibit the initial phase of ruminal UFA BH. The tendency of increased plasma PUFA and PUFA/SFA in the HCTE group is likely due to the upregulation of stearoyl coenzyme A desaturase (SCD) activity. Indeed, SCD has a substantial role in the biosynthesis of UFA by adding a double bond at the ∆-9 position of its precursor SFA [63]. c9 C18:1 is a byproduct of the fatty acid *de novo* synthesis pathway; however, it is endogenously synthesized by elongases of very long chain fatty acids (ELOVLs) and SCD, which function jointly to catalyze the transformation of C18:0 to cis C18:1 and subsequently to c9 C18:1 [64]. We observed that c9 C18:1 tends to increase, which might be caused by the action of ELOVLs and SCD. Moreover, HT reportedly increased c9 C18:1 content in milk via upregulating SCD [65]. Meanwhile, Wajner and Amaral [66] found that c9 C18:1 could be utilized as a fuel in the oxidation of fatty acids to ATP throughout the latter phases of animal growth and development. Guerreiro et al. [34] proposed that in lambs supplemented with CT extract, the initial BH phase of UFA inhibition decreases the proportion of t11 C18:1 while increasing C18 UFA. However, plasma t11 C18:1 decreased following HCTE supplementation in the current study, which was unexpected. This suggests that CTE supplementation may specifically minimize the alternative pathways of BH while HT is more efficient than CT. Moreover, this could be explained by considering that the fatty acid proportion in plasma is usually different from that in rumen, which might mask the effect of tannin on certain fatty acids in plasma. Thus, additional research is warranted to elucidate how tannins affect the blood fatty acid profile of ruminants.

It has been suggested that the fatty acid composition of meat can be regulated by diet [67]. Individuals who ingest large amounts of SFA have an increased risk of developing heart disease [68]. In contrast, UFA, especially n-3 PUFA, has been shown to have many advantages, including the ability to reduce inflammation, modulate glucolipid metabolism, and promote muscle growth [69]. Accordingly, the Food and Agriculture Organization of the United Nations has recommended diets with fewer SFA and more n-3 PUFA. The results of the current study are consistent with this recommendation. To better comprehend the effect of dietary treatment on fatty acids, nutritionists calculate the PUFA/SFA, n-6/n-3, AI, and TI based on the amount of fatty acids in tissues. The proposed upper reference limit values of AI and TI for lambs are 1.00 and 1.58, respectively [70]. In this study, the AI and TI of each longissimus dorsi muscle and subcutaneous fat were below the upper limit values. Furthermore, AI decreased linearly or quadratically, while TI tended to decrease with an increase in CTE addition. Therefore, it seems that CTE supplementation increased fatty acid unsaturation in Tan lamb tissues while lowering the risk of cardiovascular and cerebrovascular disorders. In particular, a tendency for thrombogenic acid (C16:0) to decrease was observed, while anti-thrombogenic acid (C20:5n-3) and anti-atherogenic acid (c9, t11 CLA) significantly increased. C16:0 is negatively correlated with blood LDL-C concentrations [71], whereas C20:5n-3 reduces the risk of cardiovascular disease by dilating blood vessels and enhancing immune competence [72]. Hence the observed increase in C20:5n-3 agrees with the findings of Vera et al. [73]. c9, t11 CLA is a 28 CLA isomer with high bioactivity and proportion and helps promote body health [74]. In the current study, we observed a substantial elevation in c9, t11 CLA in subcutaneous fat but no significant changes in longissimus dorsi muscle. These results might be related to lower muscle fat deposition. According to research, up to 93% of c9, t11 CLA in milk originates through t11 C18:1 conversion [9]. Meanwhile, Palmquist et al. [10] noted that the extent of c9, t11 CLA endogenous biosynthesis in lamb muscle was similar to that reported in milk. One of the effective ways to increase c9, t11 CLA in ruminant products is to favor rumen production of t11 C18:1, as it serves as the prime precursor for c9, t11 CLA synthesis via the endogenous pathway in ruminants through SCD conversion in tissues [59]. Therefore, the higher accumulation of ruminal t11 C18:1 in the CTE groups is desirable. Additionally, we discovered that the PUFA/SFA of CTE-supplemented Tan lambs ranged from 0.28 to 0.29 in longissimus dorsi muscle and 0.18 in subcutaneous fat, which were below the proposed ratio of 0.45 for the human diet [73]. It might be that the additive amount of CTE does not yet have the cumulative capacity to result in such impacts. Besides, Toshimitsu et al. [75] stated that nonalcoholic fatty liver disease might be induced in humans following consumption of animal products with n-6/n-3 ≥ 15. In the current study, the n-6/n-3 ratios in longissimus dorsi muscle and subcutaneous fat were lower than 15 in all groups. Notably, the increase in c11 C18:1 in longissimus dorsi muscle may be due to an incomplete BH process induced by the addition of CTE, in which UFA is absorbed by the intestine after being passed to the lower gastrointestinal tract and eventually deposited into the muscle.

We also observed that the fatty acid composition of the longissimus dorsi muscle and subcutaneous fat partially coincided with the results from the rumen. This demonstrates that the addition of CTE could protect UFA from BH to some extent, thereby promoting the accumulation of healthy fatty acids. However, certain inconsistencies were observed in the fatty acid profile among the longissimus dorsi muscle, subcutaneous fat, and rumen, indicating a broader influence of tannins. In particular, rumen bacteria can metabolize tannins into low molecular mass phenolics, which are absorbed via the intestine [76]. Thus, the change in fatty acid concentration could be the consequence of tannin’s actions, which does not simply act on the broad microbial community, but can reduce the number of bacteria involved in rumen BH, favoring a greater flux of healthy fatty acids to tissue sites [77].

## Conclusions

Our results indicate that CTE supplementation enhances the ADG of finishing Tan lambs in a dose-dependent manner. Moreover, dietary CTE modulates the metabolites and antioxidant capacity of plasma, facilitates rumen fermentation in Tan lambs, and may modify the fatty acid profile of the body by inducing a beneficial shift in the rumen BH pattern. Overall, CTE exhibits promising potential as a natural feed additive for ruminant production.

### Electronic supplementary material

Below is the link to the electronic supplementary material.

Supplementary Material 1

Supplementary Material 2

Supplementary Material 3

## Data Availability

The data supporting this study’s findings are available from the corresponding author upon reasonable request.

## Abbreviations

HT: hydrolyzable tannins
CT: condensed tannins
DMI: dry matter intake
CTE: chestnut tannin extract
CON: control
LCTE: low-dose CTE
HCTE: high-dose CTE
TMR: total mixed ration
VFA: volatile fatty acid
TG: triglycerides
GLU: glucose
CHOL: cholesterol
TP: total protein
ALB: albumin
LDL-C: low-density lipoprotein cholesterol
HDL-C: high-density lipoprotein cholesterol
GLB: globulin
MDA: malondialdehyde
SOD: superoxide dismutase
GSH-Px: glutathione peroxidase
T-AOC: total antioxidant capacity
IgM: immunoglobulin M
IgA: immunoglobulin A
IgG: immunoglobulin G
BW: body weight
ADG: average daily gain
FCR: feed conversion rate
BCVFA: branched-chain volatile fatty acids
PUFA: polyunsaturated fatty acids
SFA: saturated fatty acids
MUFA: monounsaturated fatty acids
AI: atherogenic index
TI: thrombogenic index
UFA: unsaturated fatty acids
CLA: conjugated linoleic acids
SCD: stearoyl coenzyme A desaturase
ELOVLs: elongases of very long chain fatty acids
BH: biohydrogenation
