# Supplementary material for: Chestnut tannin extract modulates growth performance and fatty acid composition in finishing Tan lambs by regulating blood antioxidant capacity, rumen fermentation, and biohydrogenation
Source: BMC Vet Res. 2024 Jan 10;20:23. doi: 10.1186/s12917-023-03870-3 (PMC10782739; doi:10.1186/s12917-023-03870-3)
Supplement: Supplementary file 1 — Supplementary Material 1 [file 12917_2023_3870_MOESM1_ESM.docx]

**Additional file 1:**

**Supplementary file 1.** Effect of chestnut tannin extract on the fatty acid profile of ruminal in finishing Tan lambs (g/100 g of total fatty acids)

| **Item** | **CTE Addition** | | | **SEM** | ***P*-Value** | | |
| --- | --- | --- | --- | --- | --- | --- | --- |
|  | **CON** | **LCTE** | **HCTE** |  | **G** | **L** | **Q** |
| C18:0 | 1.64 | 1.55 | 1.52 | 0.046 | 0.23 | 0.10 | 0.64 |
| C18:1 | 34.15 | 34.49 | 33.73 | 0.428 | 0.48 | 0.50 | 0.32 |
| t6 C18:1 | 0.34^b^ | 0.33^b^ | 0.42^a^ | 0.019 | <0.01 | <0.01 | 0.06 |
| t9 C18:1 | 0.55 | 0.66 | 0.72 | 0.048 | 0.08 | 0.03 | 0.70 |
| t10 C18:1 | 0.01 | 0.01 | 0.01 | 0.000 | 0.88 | 0.65 | 0.85 |
| t11 C18:1 | 8.16 | 8.40 | 8.91 | 0.231 | 0.10 | 0.04 | 0.65 |
| c9 C18:1 | 14.63 | 15.03 | 14.59 | 0.333 | 0.60 | 0.94 | 0.33 |
| c11 C18:1 | 10.49 | 10.06 | 10.30 | 0.254 | 0.51 | 0.60 | 0.31 |
| C18:2 | 26.83 | 26.19 | 26.33 | 0.414 | 0.54 | 0.41 | 0.46 |
| C18:2n-6 | 0.67 | 0.70 | 0.71 | 0.017 | 0.20 | 0.09 | 0.60 |
| t9, t12 C18:2 | 0.11 | 0.11 | 0.11 | 0.006 | 0.74 | 0.63 | 0.55 |
| t9, t13 C18:2 | 0.13 | 0.12 | 0.12 | 0.006 | 0.48 | 0.28 | 0.60 |
| c9, t12 C18:2 | 0.23 | 0.22 | 0.20 | 0.012 | 0.17 | 0.07 | 0.97 |
| t9, c12 C18:2 | 0.42^b^ | 0.45^b^ | 0.55^a^ | 0.024 | <0.01 | <0.01 | 0.30 |
| t11, c15 C18:2 | 0.25 | 0.22 | 0.22 | 0.012 | 0.12 | 0.09 | 0.20 |
| c10, t12 C18:2 | 0.03 | 0.03 | 0.03 | 0.003 | 0.50 | 0.27 | 0.72 |
| t9, c11 C18:2 | 0.07 | 0.07 | 0.07 | 0.004 | 0.91 | 0.95 | 0.67 |
| c9, t11 CLA | 0.65 | 0.66 | 0.72 | 0.035 | 0.38 | 0.20 | 0.60 |
| t10, c12 CLA | 0.43 | 0.48 | 0.51 | 0.026 | 0.11 | 0.04 | 0.68 |
| C20:5n-3 | 0.13 | 0.13 | 0.12 | 0.004 | 0.86 | 0.61 | 0.85 |
| C22:6n-3 | 0.09 | 0.09 | 0.09 | 0.004 | 0.76 | 0.60 | 0.62 |
| SFA | 1.64 | 1.55 | 1.52 | 0.046 | 0.23 | 0.10 | 0.64 |
| MUFA | 68.32 | 68.98 | 68.68 | 0.410 | 0.54 | 0.55 | 0.36 |
| PUFA | 30.04 | 29.47 | 29.80 | 0.391 | 0.60 | 0.67 | 0.37 |
| UFA | 98.36 | 98.45 | 98.48 | 0.046 | 0.23 | 0.10 | 0.64 |
| n-6 PUFA | 0.67 | 0.70 | 0.71 | 0.017 | 0.20 | 0.09 | 0.60 |
| n-3 PUFA | 0.22 | 0.22 | 0.22 | 0.006 | 0.89 | 0.97 | 0.64 |
| MUFA/SFA | 41.86 | 44.73 | 45.22 | 1.471 | 0.26 | 0.13 | 0.52 |
| PUFA/SFA | 18.40 | 19.06 | 19.62 | 0.519 | 0.29 | 0.12 | 0.94 |
| UFA/SFA | 60.27 | 63.79 | 64.83 | 1.913 | 0.25 | 0.12 | 0.61 |
| MUFA/PUFA | 2.27 | 2.35 | 2.31 | 0.045 | 0.56 | 0.62 | 0.35 |
| n-6/n-3 | 3.09 | 3.16 | 3.26 | 0.077 | 0.30 | 0.13 | 0.91 |

CTE = chestnut tannin extract; CON = control; LCTE = 2 g/kg chestnut tannin extract; HCTE = 4 g/kg chestnut tannin extract; CLA = conjugated linoleic acids; SFA = saturated fatty acids; MUFA = monounsaturated fatty acids; PUFA = polyunsaturated fatty acids; UFA = unsaturated fatty acids; n-6/n-3 = n-6 PUFA/n-3 PUFA. The effects included group (G) effects, linear (L) effects, and quadratic (Q) effects. Values are mean ± standard error of the mean (SEM). On a single line, data features distinct letters (a, b) that represent significant differences (*P* < 0.05).
