# Supplementary material for: Chestnut tannin extract modulates growth performance and fatty acid composition in finishing Tan lambs by regulating blood antioxidant capacity, rumen fermentation, and biohydrogenation
Source: BMC Vet Res. 2024 Jan 10;20:23. doi: 10.1186/s12917-023-03870-3 (PMC10782739; doi:10.1186/s12917-023-03870-3)
Supplement: Supplementary file 2 — Supplementary Material 2 [file 12917_2023_3870_MOESM2_ESM.docx]

**Additional file 2:**

**Supplementary file 2.** Effect of chestnut tannin extract on the fatty acid composition of the plasma in finishing Tan lambs (g/100 g of total fatty acids)

| **Item** | **CTE Addition** | | | **SEM** | ***P*-Value** | | |
| --- | --- | --- | --- | --- | --- | --- | --- |
|  | **CON** | **LCTE** | **HCTE** |  | **G** | **L** | **Q** |
| C14:0 | 1.20 | 1.19 | 1.20 | 0.014 | 0.87 | 0.91 | 0.61 |
| C14:1 | 0.34 | 0.35 | 0.35 | 0.008 | 0.47 | 0.28 | 0.58 |
| C15:0 | 2.09 | 2.07 | 2.02 | 0.021 | 0.10 | 0.04 | 0.70 |
| C15:1 | 6.43 | 6.49 | 6.42 | 0.038 | 0.37 | 0.96 | 0.17 |
| C16:0 | 21.96 | 21.99 | 21.94 | 0.079 | 0.93 | 0.90 | 0.73 |
| C16:1 | 11.29 | 11.26 | 11.23 | 0.046 | 0.65 | 0.37 | 0.90 |
| C17:0 | 1.62 | 1.64 | 1.64 | 0.025 | 0.77 | 0.50 | 0.81 |
| C17:1 | 1.03 | 1.02 | 1.03 | 0.008 | 0.58 | 0.94 | 0.31 |
| C18:0 | 30.63 | 30.85 | 30.75 | 0.206 | 0.75 | 0.68 | 0.53 |
| C18:1 | 11.85^a^ | 11.66^b^ | 11.61^b^ | 0.035 | <0.01 | <0.01 | 0.15 |
| t6 C18:1 | 0.10 | 0.09 | 0.09 | 0.001 | 0.28 | 0.12 | 0.91 |
| t9 C18:1 | 0.08 | 0.08 | 0.08 | 0.001 | 0.58 | 0.69 | 0.35 |
| t11 C18:1 | 1.19^a^ | 1.17^ab^ | 1.15^b^ | 0.008 | 0.03 | 0.01 | 0.63 |
| c9 C18:1 | 3.04 | 3.04 | 3.16 | 0.039 | 0.09 | 0.06 | 0.23 |
| c11 C18:1 | 0.17 | 0.17 | 0.18 | 0.006 | 0.56 | 0.41 | 0.50 |
| C18:2 | 5.22 | 5.18 | 5.35 | 0.056 | 0.12 | 0.12 | 0.15 |
| C18:2n-6 | 0.04^b^ | 0.04^b^ | 0.05^a^ | 0.001 | 0.03 | 0.01 | 0.43 |
| t9, t12 C18:2 | 0.06 | 0.06 | 0.06 | 0.001 | 0.38 | 0.23 | 0.49 |
| c9, t12 C18:2 | 0.02 | 0.02 | 0.02 | 0.001 | 0.61 | 0.45 | 0.54 |
| t9, c12 C18:2 | 0.11 | 0.11 | 0.12 | 0.002 | 0.12 | 0.06 | 0.38 |
| c9, t11 CLA | 0.23 | 0.23 | 0.24 | 0.005 | 0.11 | 0.05 | 0.44 |
| t10, c12 CLA | 0.14 | 0.15 | 0.15 | 0.003 | 0.31 | 0.13 | 0.84 |
| C18:3n-3 | 0.02 | 0.02 | 0.02 | 0.001 | 0.92 | 0.95 | 0.70 |
| C20:0 | 0.01 | 0.01 | 0.01 | 0.000 | 0.59 | 0.42 | 0.54 |
| C22:6n-3 | 0.01 | 0.01 | 0.01 | 0.000 | 0.42 | 0.32 | 0.40 |
| C24:0 | 1.14 | 1.11 | 1.11 | 0.011 | 0.15 | 0.08 | 0.38 |
| SFA | 58.63 | 58.84 | 58.66 | 0.139 | 0.53 | 0.88 | 0.27 |
| MUFA | 35.51 | 35.33 | 35.32 | 0.117 | 0.44 | 0.25 | 0.58 |
| PUFA | 5.85 | 5.82 | 6.02 | 0.059 | 0.08 | 0.07 | 0.14 |
| UFA | 41.37 | 41.16 | 41.34 | 0.139 | 0.53 | 0.88 | 0.27 |
| n-6 PUFA | 0.05^b^ | 0.05^b^ | 0.06^a^ | 0.001 | 0.01 | <0.01 | 0.37 |
| n-3 PUFA | 0.02 | 0.02 | 0.02 | 0.001 | 0.84 | 0.58 | 0.90 |
| MUFA/SFA | 0.61 | 0.60 | 0.60 | 0.003 | 0.54 | 0.45 | 0.42 |
| PUFA/SFA | 0.10 | 0.10 | 0.10 | 0.001 | 0.10 | 0.11 | 0.14 |
| UFA/SFA | 0.71 | 0.70 | 0.70 | 0.004 | 0.53 | 0.87 | 0.27 |
| MUFA/PUFA | 6.07 | 6.07 | 5.87 | 0.058 | 0.05 | 0.03 | 0.19 |
| n-6/n-3 | 2.09 | 2.13 | 2.21 | 0.061 | 0.42 | 0.20 | 0.79 |

CTE = chestnut tannin extract; CON = control; LCTE = 2 g/kg chestnut tannin extract; HCTE = 4 g/kg chestnut tannin extract; CLA = conjugated linoleic acids; SFA = saturated fatty acids; MUFA = monounsaturated fatty acids; PUFA = polyunsaturated fatty acids; UFA = unsaturated fatty acids; n-6/n-3 = n-6 PUFA/n-3 PUFA. The effects included group (G) effects, linear (L) effects, and quadratic (Q) effects. Values are mean ± standard error of the mean (SEM). On a single line, data features distinct letters (a, b) that represent significant differences (*P* < 0.05).
