# Supplementary material for: Chestnut tannin extract modulates growth performance and fatty acid composition in finishing Tan lambs by regulating blood antioxidant capacity, rumen fermentation, and biohydrogenation
Source: BMC Vet Res. 2024 Jan 10;20:23. doi: 10.1186/s12917-023-03870-3 (PMC10782739; doi:10.1186/s12917-023-03870-3)
Supplement: Supplementary file 3 — Supplementary Material 3 [file 12917_2023_3870_MOESM3_ESM.docx]

**Additional file 3:**

**Supplementary file 3.** Effect of chestnut tannin extract on the fatty acid composition of subcutaneous fat in finishing Tan lambs (g/100 g of total fatty acids)

| **Item** | **CTE Addition** | | | **SEM** | ***P*-Value** | | |
| --- | --- | --- | --- | --- | --- | --- | --- |
|  | **CON** | **LCTE** | **HCTE** |  | **G** | **L** | **Q** |
| C10:0 | 0.14 | 0.14 | 0.13 | 0.002 | 0.56 | 0.29 | 0.89 |
| C12:0 | 0.10 | 0.09 | 0.09 | 0.002 | 0.66 | 0.56 | 0.50 |
| C14:0 | 5.19 | 5.13 | 5.06 | 0.104 | 0.69 | 0.41 | 0.93 |
| C14:1 | 0.16 | 0.16 | 0.16 | 0.003 | 0.91 | 0.68 | 0.97 |
| C16:0 | 18.88 | 18.33 | 18.57 | 0.386 | 0.61 | 0.58 | 0.42 |
| C16:1 | 1.72 | 1.74 | 1.73 | 0.040 | 0.88 | 0.75 | 0.71 |
| C17:0 | 3.06 | 3.04 | 3.07 | 0.061 | 0.93 | 0.96 | 0.72 |
| C17:1 | 3.56 | 3.56 | 3.59 | 0.076 | 0.95 | 0.79 | 0.89 |
| C18:0 | 9.92 | 9.79 | 9.81 | 0.044 | 0.12 | 0.09 | 0.20 |
| t6 C18:1 | 0.08 | 0.08 | 0.08 | 0.002 | 0.49 | 0.32 | 0.53 |
| t9 C18:1 | 3.26 | 3.22 | 3.33 | 0.080 | 0.58 | 0.52 | 0.42 |
| t10 C18:1 | 9.53 | 9.43 | 9.69 | 0.185 | 0.62 | 0.55 | 0.45 |
| t11 C18:1 | 0.30 | 0.30 | 0.30 | 0.005 | 0.83 | 0.56 | 0.99 |
| t12 C18:1 | 2.65 | 2.63 | 2.63 | 0.037 | 0.89 | 0.71 | 0.78 |
| c9 C18:1 | 26.51 | 27.02 | 26.34 | 0.799 | 0.82 | 0.88 | 0.55 |
| c10 C18:1 | 0.16 | 0.16 | 0.16 | 0.003 | 0.85 | 0.58 | 0.98 |
| c11 C18:1 | 0.78 | 0.78 | 0.77 | 0.016 | 0.91 | 0.67 | 0.94 |
| c12 C18:2 | 0.15 | 0.15 | 0.15 | 0.003 | 0.93 | 0.71 | 0.98 |
| C18:2n-6 | 4.61 | 4.76 | 4.62 | 0.084 | 0.38 | 0.96 | 0.18 |
| c10, t12 C18:2 | 0.21 | 0.20 | 0.21 | 0.004 | 0.79 | 0.96 | 0.50 |
| c9, t11 CLA | 0.50^b^ | 0.52^a^ | 0.52^a^ | 0.003 | <0.01 | <0.01 | 0.05 |
| t10, c12 CLA | 0.33 | 0.34 | 0.33 | 0.008 | 0.39 | 0.62 | 0.20 |
| C18:3n-3 | 0.59 | 0.58 | 0.59 | 0.012 | 0.94 | 0.83 | 0.79 |
| C18:3n-6 | 0.87 | 0.87 | 0.87 | 0.017 | 0.99 | 0.88 | 0.99 |
| C20:0 | 6.02^c^ | 6.25^b^ | 6.48^a^ | 0.058 | <0.01 | <0.01 | 1.00 |
| C20:1 | 0.25 | 0.25 | 0.25 | 0.005 | 0.70 | 0.51 | 0.61 |
| C20:2n-6 | 0.04 | 0.04 | 0.04 | 0.001 | 0.71 | 0.43 | 0.86 |
| C20:3n-6 | 0.03 | 0.03 | 0.03 | 0.001 | 0.67 | 0.41 | 0.75 |
| C20:4n-6 | 0.14 | 0.14 | 0.14 | 0.003 | 0.79 | 0.52 | 0.86 |
| C20:5n-3 | 0.02 | 0.02 | 0.02 | 0.000 | 0.61 | 0.34 | 0.80 |
| C22:0 | 0.12 | 0.12 | 0.13 | 0.003 | 0.56 | 0.72 | 0.32 |
| C22:5n-3 | 0.02 | 0.02 | 0.02 | 0.000 | 0.62 | 0.71 | 0.37 |
| C22:6n-3 | 0.08 | 0.08 | 0.08 | 0.002 | 0.98 | 0.95 | 0.86 |
| SFA | 43.43 | 42.90 | 43.34 | 0.453 | 0.68 | 0.89 | 0.40 |
| MUFA | 48.97 | 49.33 | 49.03 | 0.508 | 0.87 | 0.94 | 0.61 |
| PUFA | 7.60 | 7.77 | 7.63 | 0.116 | 0.53 | 0.85 | 0.28 |
| UFA | 56.57 | 57.10 | 56.66 | 0.453 | 0.68 | 0.89 | 0.40 |
| n-6 PUFA | 5.69 | 5.84 | 5.70 | 0.098 | 0.49 | 0.94 | 0.25 |
| n-3 PUFA | 0.71 | 0.71 | 0.71 | 0.014 | 0.97 | 0.89 | 0.86 |
| MUFA/SFA | 1.13 | 1.15 | 1.13 | 0.024 | 0.77 | 0.91 | 0.49 |
| PUFA/SFA | 0.18 | 0.18 | 0.18 | 0.003 | 0.22 | 0.79 | 0.09 |
| UFA/SFA | 1.30 | 1.33 | 1.31 | 0.024 | 0.68 | 0.89 | 0.39 |
| MUFA/PUFA | 6.45 | 6.35 | 6.44 | 0.144 | 0.86 | 0.94 | 0.59 |
| n-6/n-3 | 8.00 | 8.27 | 8.04 | 0.106 | 0.20 | 0.79 | 0.08 |
| AI | 0.72 | 0.70 | 0.70 | 0.015 | 0.64 | 0.48 | 0.54 |
| TI | 1.15 | 1.12 | 1.13 | 0.022 | 0.56 | 0.53 | 0.39 |

CTE = chestnut tannin extract; CON = control; LCTE = 2 g/kg chestnut tannin extract; HCTE = 4 g/kg chestnut tannin extract; CLA = conjugated linoleic acids; SFA = saturated fatty acids; MUFA = monounsaturated fatty acids; PUFA = polyunsaturated fatty acids; UFA = unsaturated fatty acids; n-6/n-3 = n-6 PUFA/n-3 PUFA; AI = atherogenic index; TI = thrombogenic index. The effects included group (G) effects, linear (L) effects, and quadratic (Q) effects. Values are mean ± standard error of the mean (SEM). On a single line, data features distinct letters (a-c) that represent significant differences (*P* < 0.05).
